# An annotated high-content fluorescence microscopy dataset with Hoechst 33342-stained nuclei and manually labelled outlines

**DOI:** 10.1016/j.dib.2022.108769

**Published:** 2022-11-21

**Authors:** Malou Arvidsson, Salma Kazemi Rashed, Sonja Aits

**Affiliations:** Cell Death, Lysosomes and Artificial Intelligence Group, Department of Experimental Medical Science, Faculty of Medicine, Lund University, Sweden

**Keywords:** Instance segmentation, Fluorescence microscopy, Biomedical image analysis, High-content screening, Computer vision, Deep learning training and evaluation

## Abstract

Automated detection of cell nuclei in fluorescence microscopy images is a key task in bioimage analysis. It is essential for most types of microscopy-based high-throughput drug and genomic screening and is often required in smaller scale experiments as well. To develop and evaluate algorithms and neural networks that perform instance or semantic segmentation for detecting nuclei, high quality annotated data is essential.

Here we present a benchmarking dataset of fluorescence microscopy images with Hoechst 33342-stained nuclei together with annotations of nuclei, nuclear fragments and micronuclei. Images were randomly selected from an RNA interference screen with a modified U2OS osteosarcoma cell line, acquired on a Thermo Fischer CX7 high-content imaging system at 20x magnification. Labelling was performed by a single annotator and reviewed by a biomedical expert.

The dataset, called Aitslab-bioimaging1, contains 50 images showing over 2000 labelled nuclear objects in total, which is sufficiently large to train well-performing neural networks for instance or semantic segmentation. The dataset is split into training, development and test set for user convenience.

**Specifications Table**

**Table:** 

Subject	Computer Vision and Pattern Recognition Bioinformatics
Specific subject area	Analysis of microscopy images
Type of data	Fluorescence microscopy images (C01 and png format, grayscale) and corresponding images with annotated nuclear objects (png format, RGB) Conversion script for generating png images and readme file with instructions to run it
How the data were acquired	Images of modified U2OS cells stained with Hoechst 33342 were acquired in the blue fluorescence channel in a 4 × 4 grid with a high-content imaging fluorescence microscope (Thermo Fisher CellInsight CX7 High Content Screening Platform and the associated HCS Studio software) at 20x magnification. Outlines of nuclei, micronuclei and nuclear fragments visible in the images were manually labelled with the polygon tool of the CVAT annotation software (https://github.com/openvinotoolkit/cvat). All images were labelled by the same annotator (MA) and double-checked by a biomedical expert (SA), after which small corrections were made in some images. Annotations were saved as 24-bit rgb png image in which each nuclear object has a different, randomly assigned color.
Data format	Fluorescence microscopy images: original .C01 files and files converted to 8-bit .png format (Grayscale) Annotations: 24-bit .png format (RGB) Script: .py file with code .md file with instructions
Description of data collection	50 images were randomly selected from a dataset of thousands of images acquired in an RNA interference screen and converted from the microscope-generated .C01 format to 8-bit grayscale .png format. Images were randomly split into training, development and test set at a ratio 30:10:10.
Data source location	**Fluorescence microscopy images** • Institution: Victorian Centre for Functional Genomics (VCFG), Peter MacCallum Cancer Centre • City/Town/Region: Melbourne • Country: Australia • Latitude and longitude: -37.79845, 144.95645 **Labels** • Institution: Biomedical Centre (BMC), Lund University • City/Town/Region: Lund • Country: Sweden • Latitude and longitude: 55.71264, 13.20156
Data accessibility	The image dataset (Aitslab-bioimaging1), conversion script and readme file are available from the zenodo repository: https://doi.org/10.5281/zenodo.6657260 The script and readme file are also available on GitHub: https://github.com/Aitslab/bioimaging/tree/main/C01_conversion The CVAT annotation software (not created by us) with which the images were annotated is available from: https://github.com/openvinotoolkit/cvat
Related research article	This dataset is not connected to a specific article.

## Value of the Data

• The dataset is of use to bioimage analysts as well as academic and industry research groups who wish to automatize instance or semantic segmentation, and nuclear detection in particular.
• The dataset is sufficiently large to be used as sole training and benchmarking dataset. It can also be combined with other annotated datasets to improve and evaluate generalization of instance and semantic segmentation models and algorithms. Examples of complementary annotated fluorescence datasets are the BBBC039 dataset [1] (available from https://bbbc.broadinstitute.org/BBBC039/; accessed on Oct 28, 2022), which contains annotated fluorescence microscopy images of Hoechst-stained nuclei from cells treated with different chemical compounds and the BitDepth dataset [2] (available from https://github.com/masih4/BitDepth_NucSeg; accessed on Oct 28, 2022), which contains annotated fluorescence microscopy images of DAPI-stained nuclei from tissue sections. Examples of complementary datasets with annotated hematoxylin and eosin-stained nuclei can be found in a recent article by Mahbod et al. [3].
• As annotated datasets are extremely time-consuming to generate, few have been released so far, making the current dataset very valuable to the research community.
• The quality of the dataset is especially high as it has been annotated with the help of a senior biomedical researcher.

## 1 Objective

Nuclear detection is typically the first step in fluorescence microscopy image analysis, e.g. when counting cells, assessing protein levels and localization. To obtain high throughput and improve reproducibility this task needs to be automated with deep learning models or other algorithms that perform instance or semantic segmentation. To develop and evaluate such models or algorithms high quality manual annotations (“ground truth”) are essential. Only a few such annotated datasets have been produced and many of these show nuclei in tissue sections and/or visualized with hematoxylin and eosin staining. To complement the existing data, and facilitate the training and evaluation of deep learning models and algorithms for cell culture-based genetic and drug screens in particular, we annotated images of Hoechst 33342-stained nuclei from cultured U2OS osteosarcoma cells. Annotations were created with the help of a senior biomedical researcher to ensure high quality and avoid smaller nuclear structures being overlooked.

## 2 Data Description

### 2.1 Images

**Fig. 1:**
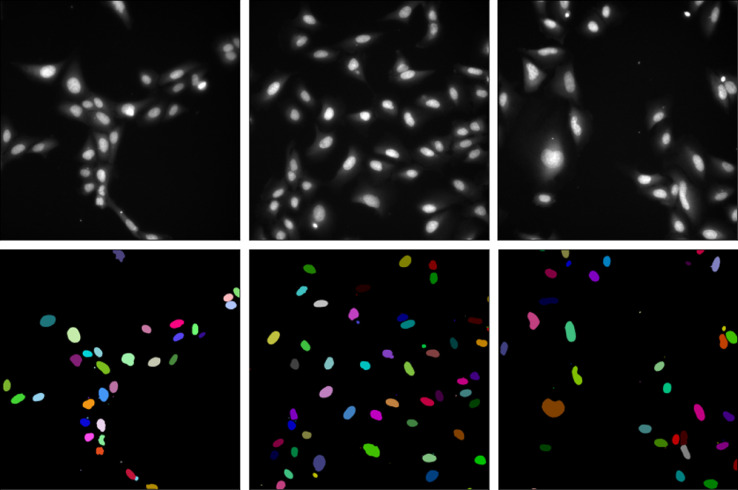
Example images of nuclei and corresponding annotations Three representative images of Hoechst 33342-stained U2OS nuclei are shown in the top row. Corresponding annotations with randomly assigned colors for each nuclear object are shown in the bottom row.

The image dataset contains 50 randomly chosen fluorescence microscopy images (original files in .C01 format and normalized images in .png format) of Hoechst 33342-stained modified U2OS cells and corresponding annotations (in .png format) of nuclei, micronuclei and nuclear fragments (examples in Fig. 1). In the annotation images, each nuclear object has a randomly assigned color to distinguish objects from each other. The dataset was split into training, development and test subset at a ratio of 30:10:10.

The file names contain a 12-digit plate ID (e.g. 170702090001), the well position (e.g. A12) and image position (e.g. f02), which refers to the position inside the well at which the image was taken, and the fluorescence channel used for imaging (d0). Annotation files have the name of the corresponding microscopy image with the suffix “_objects”.

***Training dataset***

**Table:** 

Original microscopy images	Normalized microscopy images	Annotation images

MFGTMPcx7_170702000001_B14f07d0.C01	MFGTMPcx7_170702000001_B14f07d0.png	MFGTMPcx7_170702000001_B14f07d0_objects.png
MFGTMPcx7_170702000001_D13f04d0.C01	MFGTMPcx7_170702000001_D13f04d0.png	MFGTMPcx7_170702000001_D13f04d0_objects.png
MFGTMPcx7_170702000001_F24f14d0.C01	MFGTMPcx7_170702000001_F24f14d0.png	MFGTMPcx7_170702000001_F24f14d0_objects.png
MFGTMPcx7_170702000001_G07f02d0.C01	MFGTMPcx7_170702000001_G07f02d0.png	MFGTMPcx7_170702000001_G07f02d0_objects.png
MFGTMPcx7_170702090001_A02f07d0.C01	MFGTMPcx7_170702090001_A02f07d0.png	MFGTMPcx7_170702090001_A02f07d0_objects.png
MFGTMPcx7_170702090001_A08f12d0.C01	MFGTMPcx7_170702090001_A08f12d0.png	MFGTMPcx7_170702090001_A08f12d0_objects.png
MFGTMPcx7_170702090001_A10f11d0.C01	MFGTMPcx7_170702090001_A10f11d0.png	MFGTMPcx7_170702090001_A10f11d0_objects.png
MFGTMPcx7_170702090001_A12f00d0.C01	MFGTMPcx7_170702090001_A12f00d0.png	MFGTMPcx7_170702090001_A12f00d0_objects.png
MFGTMPcx7_170702090001_B08f09d0.C01	MFGTMPcx7_170702090001_B08f09d0.png	MFGTMPcx7_170702090001_B08f09d0_objects.png
MFGTMPcx7_170702090001_C09f01d0.C01	MFGTMPcx7_170702090001_C09f01d0.png	MFGTMPcx7_170702090001_C09f01d0_objects.png
MFGTMPcx7_170702090001_C16f04d0.C01	MFGTMPcx7_170702090001_C16f04d0.png	MFGTMPcx7_170702090001_C16f04d0_objects.png
MFGTMPcx7_170702090001_F20f14d0.C01	MFGTMPcx7_170702090001_F20f14d0.png	MFGTMPcx7_170702090001_F20f14d0_objects.png
MFGTMPcx7_170702090001_H03f10d0.C01	MFGTMPcx7_170702090001_H03f10d0.png	MFGTMPcx7_170702090001_H03f10d0_objects.png
MFGTMPcx7_170702090001_K22f04d0.C01	MFGTMPcx7_170702090001_K22f04d0.png	MFGTMPcx7_170702090001_K22f04d0_objects.png
MFGTMPcx7_170702090001_L21f03d0.C01	MFGTMPcx7_170702090001_L21f03d0.png	MFGTMPcx7_170702090001_L21f03d0_objects.png
MFGTMPcx7_170702090001_N06f14d0.C01	MFGTMPcx7_170702090001_N06f14d0.png	MFGTMPcx7_170702090001_N06f14d0_objects.png
MFGTMPcx7_170702090001_O02f15d0.C01	MFGTMPcx7_170702090001_O02f15d0.png	MFGTMPcx7_170702090001_O02f15d0_objects.png
MFGTMPcx7_170702090001_P08f09d0.C01	MFGTMPcx7_170702090001_P08f09d0.png	MFGTMPcx7_170702090001_P08f09d0_objects.png
MFGTMPcx7_170731090001_B05f10d0.C01	MFGTMPcx7_170731090001_B05f10d0.png	MFGTMPcx7_170731090001_B05f10d0_objects.png
MFGTMPcx7_170731090001_B14f13d0.C01	MFGTMPcx7_170731090001_B14f13d0.png	MFGTMPcx7_170731090001_B14f13d0_objects.png
MFGTMPcx7_170731090001_G15f00d0.C01	MFGTMPcx7_170731090001_G15f00d0.png	MFGTMPcx7_170731090001_G15f00d0_objects.png
MFGTMPcx7_170731090001_G15f03d0.C01	MFGTMPcx7_170731090001_G15f03d0.png	MFGTMPcx7_170731090001_G15f03d0_objects.png
MFGTMPcx7_170731090001_I12f02d0.C01	MFGTMPcx7_170731090001_I12f02d0.png	MFGTMPcx7_170731090001_I12f02d0_objects.png
MFGTMPcx7_170731090001_I12f07d0.C01	MFGTMPcx7_170731090001_I12f07d0.png	MFGTMPcx7_170731090001_I12f07d0_objects.png
MFGTMPcx7_170731090001_K05f07d0.C01	MFGTMPcx7_170731090001_K05f07d0.png	MFGTMPcx7_170731090001_K05f07d0_objects.png
MFGTMPcx7_170731090001_K24f09d0.C01	MFGTMPcx7_170731090001_K24f09d0.png	MFGTMPcx7_170731090001_K24f09d0_objects.png
MFGTMPcx7_170731090001_K24f10d0.C01	MFGTMPcx7_170731090001_K24f10d0.png	MFGTMPcx7_170731090001_K24f10d0_objects.png
MFGTMPcx7_170801050001_A01f03d0.C01	MFGTMPcx7_170801050001_A01f03d0.png	MFGTMPcx7_170801050001_A01f03d0_objects.png
MFGTMPcx7_170802000001_I12f01d0.C01	MFGTMPcx7_170802000001_I12f01d0.png	MFGTMPcx7_170802000001_I12f01d0_objects.png
MFGTMPcx7_170803210001_P17f28d0.C01	MFGTMPcx7_170803210001_P17f28d0.png	MFGTMPcx7_170803210001_P17f28d0_objects.png

***Development dataset***

**Table:** 

Original microscopy images	Normalized microscopy images	Annotation images

MFGTMPcx7_170702000001_B23f07d0.C01	MFGTMPcx7_170702000001_B23f07d0.png	MFGTMPcx7_170702000001_B23f07d0_objects.png
MFGTMPcx7_170702000001_F11f10d0.C01	MFGTMPcx7_170702000001_F11f10d0.png	MFGTMPcx7_170702000001_F11f10d0_objects.png
MFGTMPcx7_170702090001_A08f09d0.C01	MFGTMPcx7_170702090001_A08f09d0.png	MFGTMPcx7_170702090001_A08f09d0_objects.png
MFGTMPcx7_170702090001_A20f02d0.C01	MFGTMPcx7_170702090001_A20f02d0.png	MFGTMPcx7_170702090001_A20f02d0_objects.png
MFGTMPcx7_170702090001_G03f02d0.C01	MFGTMPcx7_170702090001_G03f02d0.png	MFGTMPcx7_170702090001_G03f02d0_objects.png
MFGTMPcx7_170702090001_K22f14d0.C01	MFGTMPcx7_170702090001_K22f14d0.png	MFGTMPcx7_170702090001_K22f14d0_objects.png
MFGTMPcx7_170702090001_P01f02d0.C01	MFGTMPcx7_170702090001_P01f02d0.png	MFGTMPcx7_170702090001_P01f02d0_objects.png
MFGTMPcx7_170731090001_A01f04d0.C01	MFGTMPcx7_170731090001_A01f04d0.png	MFGTMPcx7_170731090001_A01f04d0_objects.png
MFGTMPcx7_170731090001_B05f12d0.C01	MFGTMPcx7_170731090001_B05f12d0.png	MFGTMPcx7_170731090001_B05f12d0_objects.png
MFGTMPcx7_170802000001_I10f05d0.C01	MFGTMPcx7_170802000001_I10f05d0.png	MFGTMPcx7_170802000001_I10f05d0_objects.png

***Test dataset***

**Table:** 

Original microscopy images	Normalized microscopy images	Annotation images

MFGTMPcx7_170702000001_G14f03d0.C01	MFGTMPcx7_170702000001_G14f03d0.png	MFGTMPcx7_170702000001_G14f03d0_objects.png
MFGTMPcx7_170702090001_B22f15d0.C01	MFGTMPcx7_170702090001_B22f15d0.png	MFGTMPcx7_170702090001_B22f15d0_objects.png
MFGTMPcx7_170702090001_C08f14d0.C01	MFGTMPcx7_170702090001_C08f14d0.png	MFGTMPcx7_170702090001_C08f14d0_objects.png
MFGTMPcx7_170702090001_H04f01d0.C01	MFGTMPcx7_170702090001_H04f01d0.png	MFGTMPcx7_170702090001_H04f01d0_objects.png
MFGTMPcx7_170702090001_P07f14d0.C01	MFGTMPcx7_170702090001_P07f14d0.png	MFGTMPcx7_170702090001_P07f14d0_objects.png
MFGTMPcx7_170731090001_B14f09d0.C01	MFGTMPcx7_170731090001_B14f09d0.png	MFGTMPcx7_170731090001_B14f09d0_objects.png
MFGTMPcx7_170731090001_D11f13d0.C01	MFGTMPcx7_170731090001_D11f13d0.png	MFGTMPcx7_170731090001_D11f13d0_objects.png
MFGTMPcx7_170731090001_I12f05d0.C01	MFGTMPcx7_170731090001_I12f05d0.png	MFGTMPcx7_170731090001_I12f05d0_objects.png
MFGTMPcx7_170801050001_G02f01d0.C01	MFGTMPcx7_170801050001_G02f01d0.png	MFGTMPcx7_170801050001_G02f01d0_objects.png
MFGTMPcx7_170803210001_J12f29d0.C01	MFGTMPcx7_170803210001_J12f29d0.png	MFGTMPcx7_170803210001_J12f29d0_objects.png

### 2.2 Script

C01_to_png.py contains the script used to generate the normalized .png fluorescence microscopy images from the original .C01 files.

Readme.md contains instructions for running the script.

## 3 Experimental Design, Materials and Methods

Modified U2OS cells were plated in black clear-bottom 384-well plates (Greiner) and transfected with siRNAs (Dharmacon siGENOME library), which were washed away the next day. After 72 h, cells were fixed and stained simultaneously with Hoechst 33342. Plates were stored sealed at 4 degrees Celsius until imaging in a CX7 high-content imaging system (Thermo Fisher). For each well, 16 images were acquired in a non-overlapping grid at 20x magnification in the blue fluorescence channel using the microscope-associated HCS Studio software.

50 images, derived from multiple 384-well plates, were randomly chosen for annotation. Images were exported in the microscope-generated .C01 format. Prior to annotation, .C01 images were normalized and transformed to 8-bit png images using the C01_to_png.py script (available from https://github.com/Aitslab/bioimaging/tree/main/C01_conversion) as follows:

1. Download and install bftools:

cd∼/bin

wget http://downloads.openmicroscopy.org/latest/bio-formats/artifacts/bftools.zip

unzip bftools.zip

rm bftools.zip

export PATH=$PATH:∼/bin/bftools

2. Install required python packages:

pip install argparse

pip install os

pip install subprocess

pip install tqdm

pip install pathlib

pip install numpy

pip install scikit-image

3. Perform .C01 to .tiff conversion and subsequent normalization and .png conversion with C01_to_png.py script:

python3 C01_to_png.py -i INDIR -o OUTDIR -ift C01 -oft tiff

Nuclei, nuclear fragments and micronuclei visible in the png images were annotated as a single class with the polygon tool of the CVAT annotation software (https://github.com/openvinotoolkit/cvat). Annotations were made by a single trained researcher (MA) and double-checked by a senior biomedical expert (SA), after which small corrections were made in some images. Annotations were saved as 24-bit rgb png image with each nuclear object filled in with a randomly assigned different color.

## Ethics statements

A commercially available human cancer cell line was used for these studies. Therefore, no ethical permits were required.

## CRediT Author Statement

**Sonja Aits:** Conceptualization, Methodology, Validation, Investigation, Resources, Data curation, Writing, Supervision (lead), Project administration, Funding acquisition, Visualization **Malou Arvidsson:** Investigation, Methodology, Software **Salma Kazemi Rashed:** Software, Supervision (supporting).

## Declaration of Competing Interest

The authors declare that they have no known competing financial interests or personal relationships that could have appeared to influence the work reported in this paper.

## Data Availability

An annotated high-content fluorescence microscopy dataset with Hoechst 33342-stained nuclei and manually labelled outlines (Original data) (zenodo).
